# Niuhuang Qingxin Wan ameliorates depressive-like behaviors and improves hippocampal neurogenesis through modulating TrkB/ERK/CREB signaling pathway in chronic restraint stress or corticosterone challenge mice

**DOI:** 10.3389/fphar.2023.1274343

**Published:** 2024-01-11

**Authors:** Qiaohui Du, Chong Gao, Bun Tsoi, Meiling Wu, Jiangang Shen

**Affiliations:** ^1^ School of Chinese Medicine, The University of Hong Kong, Pokfulam, Hong Kong, China; ^2^ The Institute of Brain and Cognitive Sciences, School of Medicine, Zhejiang University City College, Hangzhou, China; ^3^ Department of Food Science and Nutrition, The Hong Kong Polytechnic University, Kowloon, Hong Kong, China

**Keywords:** Niuhuang Qingxin Wan, depression, hippocampal neurogenesis, neural stem cells, BDNF/TrkB/ERK/CREB pathway

## Abstract

**Introduction:** Chronic stress-associated hormonal imbalance impairs hippocampal neurogenesis, contributing to depressive and anxiety behaviors. Targeting neurogenesis is thus a promising antidepressant therapeutic strategy. Niuhuang Qingxin Wan (NHQXW) is an herbal formula for mental disorders in Traditional Chinese Medicine (TCM) practice, but its anti-depressant efficacies and mechanisms remain unverified.

**Methods:** In the present study, we tested the hypothesis that NHQXW could ameliorate depressive-like behaviors and improve hippocampal neurogenesis by modulating the TrkB/ERK/CREB signaling pathway by utilizing two depression mouse models including a chronic restraint stress (CRS) mouse model and a chronic corticosterone (CORT) stress (CCS) induced mouse model. The depression-like mouse models were orally treated with NHQXW whereas fluoxetine was used as the positive control group. We evaluated the effects of NHQXW on depressive- and anxiety-like behaviors and determined the effects of NHQXW on inducing hippocampal neurogenesis.

**Results:** NHQXW treatment significantly ameliorated depressive-like behaviors in those chronic stress mouse models. NHQXW significantly improved hippocampal neurogenesis in the CRS mice and CCS mice. The potential neurogenic mechanism of NHQXW was identified by regulating the expression levels of BDNF, TrkB, p-ERK (T202/T204), p-MEK1/2 (S217/221), and p-CREB (S133) in the hippocampus area of the CCS mice. NHQXW revealed its antidepressant and neurogenic effects that were similar to fluoxetine. Moreover, NHQXW treatment revealed long-term effects on preventing withdrawal-associated rebound symptoms in the CCS mice. Furthermore, in a bioactivity-guided quality control study, liquiritin was identified as one of the bioactive compounds of NHQXW with the bioactivities of neurogenesis-promoting effects.

**Discussion:** Taken together, NHQXW could be a promising TCM formula to attenuate depressive- and anxiety-like behaviors against chronic stress and depression. The underlying anti-depressant mechanisms could be correlated with its neurogenic activities by stimulating the TrkB/ERK/CREB signaling pathway.

## 1 Introduction

Depression is a high-prevalence chronic mental disorder worldwide (James et al., 2018). Depression patients suffer from anxiety, tiredness, hopelessness, excessive guilt, insomnia, irritability, and even suicidal tendencies (Haenisch and Bönisch, 2011). Recently, the COVID-19 outbreak led to a 7 times higher prevalence of depression than that estimated in the period before COVID-19 (Bueno-Notivol et al., 2021). Inhibiting serotonin reuptake, monoamine oxidase, pindolol, and electroconvulsive therapy are the major therapeutic approaches to ameliorate depressive symptoms (Trivedi et al., 2006; Barbui et al., 2007). However, unsatisfactory efficiencies and side effects blight their use for depression treatment (Predictable et al., 2006).

Targeting adult neurogenesis becomes a promising antidepressant therapeutic strategy (Pascual-Brazo et al., 2014). In the adult brain, the subventricular zone and subgranular zone in the dentate gyrus (DG) are two special areas that have spontaneous neurogenesis (Lois and Alvarez-Buylla, 1994; Eriksson et al., 1998). Particularly, the hippocampus is one of the major regions controlling learning, memory, and emotional behaviors (Girardeau et al., 2017). In the hippocampal DG, neural stem cells or neural progenitor cells have the capacity for proliferation and lineage commitment, eventually developing into mature neurons and integrating into neural circuits. Impaired neurogenesis in the SGZ region has been found in patients with depression (Krogh et al., 2014; Gandy et al., 2017). Several signaling pathways including Notch signaling, Wnt/β-catenin signaling, and sonic hedgehog signaling (SHH); growth factors; neurotrophic factors; and neurotransmitters play a vital role in neurogenesis (Bond et al., 2012; Faigle and Song, 2013; Liu et al., 2018; Wagenaar et al., 2018; Ho et al., 2020; Xu et al., 2020). For example, chronic stress inhibits BDNF secretion and reduces its downstream signaling cascades including the activities of tyrosin kinase receptor TrkB and cAMP response element-binding protein (CREB) (Luo et al., 2017; Peng et al., 2018). Chronic stress also triggers hormone responses and inhibits the proliferation and differentiation of neural stem/progenitor cells (NSCs), contributing to depressive behaviors (Morais et al., 2014; Egeland et al., 2015). The effectiveness of serotonin reuptake and tricyclic antidepressant treatments are associated with the induction of the proliferation and differentiation of NSCs (Perera et al., 2007). Antidepressant treatment could elevate BDNF expression, and promote hippocampal neurogenesis and maturation of NSCs (Masi and Brovedani, 2011; Ma et al., 2017). Thus, the promotion of adult neurogenesis could be an important therapeutic strategy for antidepressant treatment.

Traditional Chinese medicine (TCM) has a long history of use for depressive and mental disorder treatment. For example, Xiaoyao San is a classic TCM formula used for anti-depressive treatment. A recent study revealed that modified Xiaoyao San (MXYS) alleviated depressive-like behaviors in a depression mouse model with chronic unpredictable mild stress by improving blood oxygen concentration and inducing hippocampal neurogenesis (Gao L. et al., 2018). Recently, we identified several natural compounds from medicinal plants with neurogenic, antidepressant, and anxiolytic effects via regulating multiple signaling pathways such as glucocorticoid receptor activity, BDNF/Akt signaling pathway, and CREB signaling pathway (Gao C. et al., 2018; He et al., 2021). Niuhuang Qingxin Wan (NHQXW) is a commonly used herbal formula in TCM practice with the potential to clinically treat mental disorder-related symptoms, including insomnia, anxiety, and irritability, in China (DUAN et al., 2018; Xiaoying, 2021; Xi, 2023). Several *in vitro* and *in vivo* experiments indicated the potential neurogenic effects of several active compounds and herbal extracts (Wang et al., 2020; Cheng et al., 2021; Tang et al., 2021; Tong et al., 2023). Therefore, in the present study, we tested the hypothesis that NHQXW could ameliorate depressive-like behaviors and improve hippocampal neurogenesis by modulating TrkB/ERK/CREB signaling pathway by utilizing two depression animal models. Given that NHQXW is a TCM formula containing many chemical ingredients, quality control study is crucial for batch-to-batch consistency. Thus, we performed a quality control study and liquiritin was identified as one of the bioactive compounds of NHQXW with neurogenesis-promoting effects.

## 2 Materials and methods

### 2.1 NHQXW extraction

NHQXW was provided by Beijing Tong Ren Tang Chinese Medicine Co. Ltd. NHQXW is composed of 27 herbal medicines including artificial Niuhuang, artificial antelope hom, artificial forest musk abelmosk, *panax ginseng* radix, *atractylodes macrocephala* radix, *angelicae sinensis* radix, *paeoniae* radix, *chuanxiong* rhizoma, *bupleuri* radix, *glycyrrhizae* radix, *smilacis glabrae rhizoma*, *platycodonis* radix, *saposhnikoviae* radix, *asini corii colla*, *cinnamomi* cortex, *ophiopogonis* radix, *ampelopsis* radix, *borneolum syntheticum*, *typhae pollen, armeniacae semen* amarum, *Jujubae* fructus, *sojae semen praeparatum*, *dioscoreae* rhizoma, *scutellariae* radix, *bubali cornu,* and *zingiberis* rhizoma. NHQXW was made with GMP standard and extracted by methanol for quality control analysis. To obtain a powder for treatment or quality control purposes, NHQXW was blended. The methanol concentration for extraction was set at 85%. The ultrasonic extraction method was applied for active compound dissolution using an ultrasonic bath (Kunshan Ultrasonic Instrument Co. Ltd., China). The condition of ultrasonic extraction was set at 30 min under room temperature with power at 150 W. For quality control, we analyzed the content of bilirubin, a representative component of artificial Niuhuang, by using an oxalic acid solution and methylene chloride under ultrasonic conditions. These extraction processes were repeated in two cycles. After centrifugation, the supernatant was filtered before LC analysis.

### 2.2 Quality control study

Quality control analysis was conducted by using a high-performance liquid chromatography (HPLC) system (ThermoFisher Scientific, UltiMate 3000) according to our previous studies (Du et al., 2020). The compound separation was performed by using the ACE 5 AQ-C18 column (5 μm, 4.6 mm × 250 mm, Advanced Chromatography Technologies). We selected liquiritin, baicalin, paeoniflorin, and bilirubin for quality control study according to Chinese Pharmacopoeia and the reported neurogenic bioactivities (Zhao et al., 2008; Fan et al., 2019; Wang et al., 2021). For detecting liquiritin, baicalin, and paeoniflorin, we used the mobile phase condition optimized as follows: 0–8 min, 19% acetonitrile (ACN); 8–35 min, 19%–50% ACN; and 35–36 min, 50%–100% ACN. The water phase contained 0.085% phosphoric acid (H_3_PO_4_). For analysis of bilirubin, the water phase was 90% ACN containing 0.1% formic acid and 10% water with 1% acetic acid for 20 min. Column temperature and sample temperature were kept at 35°C and 20 C, respectively. Each injection volume per test was 10 µL. The detector ultraviolet (UV) wavelength was set at 283 nm for the detection of liquiritin, baicalin, and paeoniflorin and 450 nm for bilirubin, and the scan range for DAD was 190–450 nm. To build calibration curves for quantification, we dissolved standards with 90% methanol at 1 mg/mL, and the standard mixture was mixed at 0.33 mg/mL for each compound. The standard solution of bilirubin was prepared at 120 μg/ml as the maximum concentration. The standard curves were established by using standard solutions with standard protocols.

### 2.3 Animal models

C57BL/6N mice were purchased from the Centre for Comparative Medicine Research, the University of Hong Kong with ages of 6–8 weeks. The animal experiment and process were approved by the University Committee on the Use of Live Animals in Teaching and Research (CULATR no. 4001-16). Mice were given *ad libitum* access to food and water.

#### 2.3.1 Chronic restraint stress (CRS)

We adapted a CRS mouse model to assess the effectiveness of NHQXW in alleviating depression-like behaviors by using the protocol as previously described (Zafir et al., 2009). In brief, the mice were placed into a 50 mL breathable falcon tube for 6 h per day which was performed for 30 days each day from 12:00 p.m. to 6:00 p.m. All the mice were restrained from physical movement without pain.

#### 2.3.2 Chronic CORT stress (CCS)

We also employed a corticosterone (CORT, Sigma-Aldrich) induced depression-like mouse model to further evaluate the anti-depressant effects by following the protocol outlined in our previous report (Gao C. et al., 2018). In brief, CORT was dissolved in drinking water with 0.45% β-cyclodextrin (Sigma-Aldrich) at the concentration of 70 μg/mL. The mice were allowed to freely take water. To avoid the taste of the CORT-containing water influencing the drinking volume, we calculated the volume of water consumption every week, and the concentration of CORT was equivalent to 5 mg/kg/day for 40 consecutive days to establish a CORT-induced depressive mouse model.

#### 2.3.3 Drug treatment

Adult male C57BL/6N mice were used for both the CRS mouse model and CORT induced depression-like mouse model. The mice were randomly divided into the following groups: normal control, stress group, stress plus NHQXW group, and stress plus fluoxetine group. The experimental models and treatment protocols are shown in Figures 2A, 3A. In the restraint stress experiments, the NHQXW solution (20 mg/g/day) was orally administrated at the time prior to the induction of restraint stress every day. In the CORT stress experiments, the NHQXW powder was dissolved into distilled water and a dosage of 20 mg/g per day of NHQXW was orally administered to the mice at day 15 after being exposed to CORT treatment (5 mg/kg/day) for 25 consecutive days. Distilled water was used as vehicle treatment which was orally administered to the vehicle-treated CORT stress mice. Fluoxetine (18 mg/kg/day) was used as the positive control group with the same procedure as NHQXW treatment in both restraint stress and CORT stress. As for the therapeutic treatment, the NHQXW was administered to the mice after 15 days of CORT exposure (D0) until the end of stress. The first day of restraint stress or CORT stress was defined as D0. Drug treatment and restraint stress were repeat every 24 h. The withdrawal protocol of NHQXW treatment was designed to stop administration after 14 days of treatment from D15 to D28 in the processing of CORT stress.

#### 2.3.4 Bromodeoxyuridine (BrdU) tracking

For tracking newborn NSCs, bromodeoxyuridine (5-bromo-2′-deoxyuridine, BrdU, Abcam) was intraperitoneally injected into the mice for 5 consecutive days at a dosage of 50 mg/kg once per day as in our previous works (Du et al., 2021). In detail, BrdU was dissolved in prewarmed distilled saline at a content of 10 mg/mL. The injection volume was modified by the body weight of the mice.

### 2.4 Behavioral tests

Several behavioral tests were applied to assess the emotional depressive- and anxiety-like behaviors, including the tail suspension test (TST), forced swimming test (FST), and open field test (OFT) as in our previous work (Du et al., 2021). The behavior tests were performed 24 h after the last treatment. The TST and FST were performed in the morning and the interval of each mouse between different tests was 30 min. Briefly, in the TST, the mouse tail was suspended from the top of the box for 6 min to record the behavior. In the whole test, we recorded the immobility time to evaluate the learned helplessness of mice. In the FST, the mice were placed into a water tank (30 cm × 20 cm) for 6 min. The temperature was kept at 23°C∼25°C. The mobility time was recorded in a cylinder filled with water. The activations including strong body shaking, movement of the limbs, and trying to reach the wall were captured. The moving time of the mice in the FST was recorded as the mobility time. In the FST, the first 2 min was recognized as habituation, and the mobility time in the last 4 min was applied for the analysis (Gao C. et al., 2018). In the OFT, the mice were placed into a transparent Plexiglas cube with the size of 43 cm × 43 cm with a center zone (20 cm × 20 cm). In the test session, the mice were placed in the corner and allowed 2 min to recover from handling. The open field would be cleaned with 75% ethanol before the test. The movement was recorded for 8 min using video tracking software. In the sucrose splash test (SST), 10% sucrose solution was used to spray on the back of the mice. The grooming time, including licking, biting, or scratching, was calculated over a 10-min period. The novelty-suppressed feeding test (NSFT) was conducted the next day after 24 h of food deprivation. NSFT was performed in an open field with a single food pellet in the center of the arena. The weight of the food was recorded to calculate the food consumption. The latency of feeding and the consumption weight of the food pellet were recorded over 10 min. All the videos were recorded with video tracking software Smart 3.0 system (RWD, Delawares).

### 2.5 Cell culture

Human embryonic stem cells (hESCs, WiCell) were cultured with the protocols described in our previous study (Tsoi et al., 2022). In brief, H7 cells were cultured in a Matrigel-coated 6-well plate (Corning). The culture medium was feeder-free TeSR-E8 medium. At the first 24 h of H7 seeding, Rho-kinase inhibitors were supplemented into the culture medium. After that, the culture medium was changed into fresh medium for every 24 h. NSCs were induced from hESCs after 2–3 days of recovery. The induction medium consisted of two separated mediums, the first of which was N2, a non-essential amino acid, and GlutaMax in DMEM/F12 medium. The other medium was a NeuroBasal medium supplemented with B27, and two SMAD inhibitors: SB431542 and LDN193189 (Watanabe et al., 2005; Chambers et al., 2009). Baicalin, paeoniflorin, and liquiritin (Pusi BioTech), three representative compounds, were prepared in DMSO at a concentration of 1 mg/ml as the stock solution. For the treatment, these compounds were diluted to 10 μg/ml. As liquiritin revealed its neurogenesis-promising effects, we tested whether the bioactivity is related to modulating TrkB signaling. The cells were co-treated with liquiritin and a TrkB selective inhibitor cyclotraxin B (Cyc-b, MCE) at a dosage of 100 nM. The control group was treated with the same volume of DMSO as the vehicle control.

### 2.6 Immunofluorescence

For immunofluorescence staining, brain samples from different groups were post-fixed with 4% PFA for 48 h, followed by dehydration in 30% sucrose solution at 4°C. After full dehydration, the brain tissues were embedded in O.C.T. and stocked in −80°C till to use. The entire length of the DG was cut into 30 μm sections as frozen slices and stored at −20°C. For *in vivo* NSCs imaging, the tissue sections were attached to Poly-D-Lysine coated microscope slides (0111500; GmbH and Co. KG). Antigen retrieval was conducted by using citrate acid buffer (pH 6.0) at boiling conditions. After a PBS wash, the brain slices were permeabilized using PBS containing 1% Triton X-100 (PBST) and blocked by 5% goat serum for 1 h at room temperature. As for the BrdU staining, the samples were incubated with 2 N HCl for 30 min followed by 0.1 M borate buffer (pH 8.5) neutralizing. The samples were incubated with primary antibodies including anti-BrdU (Abcam, 1:200), anti-doublecortin (DCX, Cell Signaling Tech, 1:400), anti-NeuN (Cell Signaling Tech, 1:400), anti-MAP2 (Cell Signaling Tech, 1:400) and anti-β Tubulin (Tuj1, Santa Cruz, 1:400). The sections were then stained with fluorochrome-conjugated secondary antibodies, and the nucleus was counterstained with DAPI and mounted with antifade medium (Dako). The fluorescent images were obtained by a confocal microscope (Zeiss LSM 800, Core facility in LSK Faculty of Medicine, HKU) and analyzed by Zeiss software. As for *in vitro* NSCs imaging, the NSCs were seeded to the Matrixgel coated slides at a density of 50,000 cells/well before 3 days of imaging. The processing methods of slides imaging were similar to methodology in the tissue section. To analyze the positive fluorescent results quantitatively, after obtaining the confocal image with z-stack, we adopted the method named “maximum intensity project” to convert the 3D image to a 2D image with an increased intensity for calculation (Du et al., 2023). Then, we calculated the BrdU/DCX or BrdU/NeuN dual positive cells colocalized with the nucleus as newborn immature or mature neurons in the brain section. To avoid an individual difference in cell counting, the positively stained cells were calculated by the authors, who had no information, in a group setting to avoid personal bias.

### 2.7 Western blot analysis

As in our previous works, the expression levels of targeted proteins were analyzed by Western blot (Du et al., 2021; Gao et al., 2022). The proteins were extracted from the hippocampal zone of the ischemic brain site by using radioimmunoprecipitation (RIPA) buffer plus with 1% protease and phosphatase inhibitor cocktails (Cell Signaling Tech). A protein assay kit (Thermo Fisher Scientific) was used for quantification of the loading contents of the protein samples. The protein samples (20 μg per well) were separated by sodium dodecyl sulfate-polyacrylamide (SDS-PAGE) gel electrophoresis and transferred on 0.45 µM polyvinylidene fluoride (PVDF) membranes for blotting. The membrane was blocked by 5% bovine serum albumin solution for 1 h at room temperature in a rotation and immunoblotted with primary antibodies following HRP-conjugated secondary antibodies. The primary antibodies applied for the blotting analysis were listed as follows: BDNF (abcam, ab108319, 1:1000), TrkB (Cell Signaling Tech, 4603, 1:1000), Phospho-MEK1/2 (S217/221) (Cell Signaling Techn, 9121, 1:1000), MEK1/2 (Cell Signaling Tech, 9122, 1:1000), Phospho-P44/P42 ERK (T202/T204) (Cell Signaling Tech, 4370, 1:1000), P44/P42 ERK (Cell Signaling Tech, 4695, 1:1000), CREB (Cell Signaling Tech, 4820, 1:1000), Phospho-CREB (S133) (Cell Signaling Tech, 9198, 1:1000), and β-Actin (Cell Signaling Tech, 3700, 1:3000). After incubation with these primary antibodies, the samples were incubated with the secondary antibodies overnight at 4°C with rotation. The membranes were visualized using a chemiluminescent ECL select kit (GE Healthcare) detected by a Gel Doc system (Bio-Rad) and analyzed using Image Lab software (Bio-Rad). The relative expression levels of BDNF and TrkB were calculated by dividing their respective volumes by the volume of β-Actin. For the phosphorylated proteins, the relative expression levels were determined by comparing the volume of phosphorylated proteins to the volume of total proteins on the same membrane. For the calculation of p-MEK1/2/T-MEK1/2, the volume of p-MEK1/2 (S217/221) was quantified in phosphorylation compared to the volume of total MEK1/2. The volume of proteins was calculated using Image Lab software (Bio-Rad).

### 2.8 Flow cytometry analysis

After NSCs induction and drug treatment for 10 days, the cells were dissociated by using Accutase (Gibco) and the isolated cells were fixed with 4% PFA for 15 min, followed conjugated fluorochromes staining overnight, including PE anti-Nestin (Biolegend, 1:200), APC anti-Tubulin *β* 3 (Biolegend, 1:200) and anti-rabbit DCX (Cell Signaling Tech, 1:2000). Alexa Flour 488 anti-rabbit secondary antibody (Thermo Fisher Scientific, 1:2000) was used to conjugate DCX for flow cytometry analysis. We detected the conjugated fluorochromes using a NovoCyte Quanteon flow cytometer (Agilent, Core facility in LSK Faculty of Medicine, HKU) for data collection, and data analysis was performed using FlowJo software.

### 2.9 Statistical analysis

All the data were presented as mean ± S.E.M. and analyzed using Prism 8 (GraphPad). Statistical analysis was performed using one-way ANOVA followed by Dunnett’s multiple-comparison test for multiple group comparisons. For the behavioral results of the animal studies, the samples were collected from two separate batches of mice experiments. The results of the Western blot were conducted with 5–6 samples from each group and repeated twice. The *in vitro* experiments were performed with three biological replicates and repeated twice. The sample sizes, degree of freedom, F values, and *p*-values were provided. The normal control group was the mice without model establishment, and it was compared to the vehicle-treated depression model group to provide information on successful model establishment. The vehicle-treated depression model was compared with the NHQXW treated depression mice to give statistics information between the treatment group and the depression group. The *p-*value of <0.05 was recognized as statistical significance.

## 3 Results

### 3.1 Quality control of NHQXW

**FIGURE 1:**
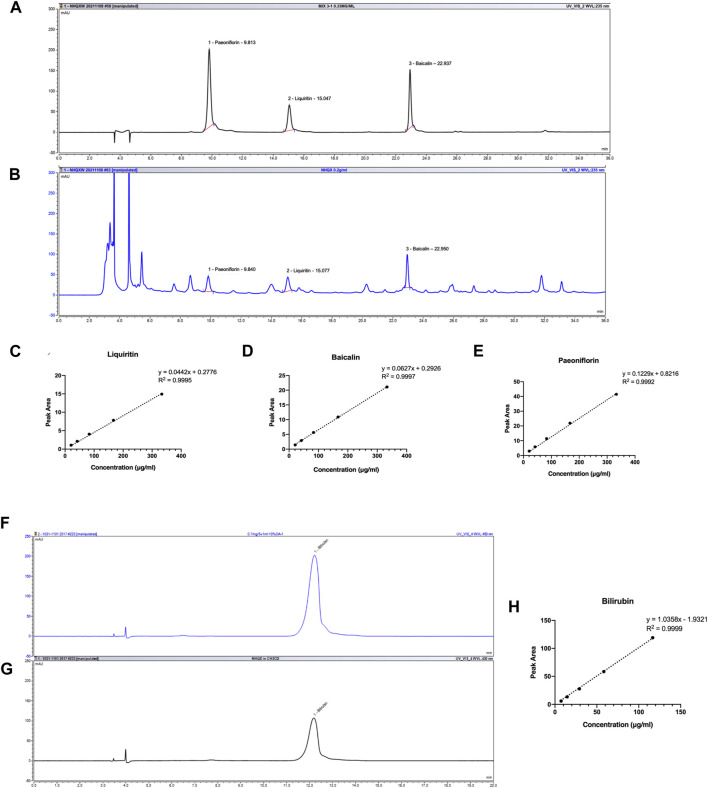
HPLC profile of NHQXW and standard mixture. **(A)** HPLC chromatography of standard mixture with detection wavelength at 235 nm. **(B)** HPLC chromatography of NHQXW extract with detection wavelength at 235 nm. **(C)** The standard curve of liquiritin by the HPLC method. **(D)** The standard curve of baicalin by the HPLC method. **(E)** The standard curve of paeoniflorin by the HPLC method. **(F)** HPLC chromatography of bilirubin with detection wavelength at 450 nm. **(G)** HPLC chromatography of bilirubin in NHQXW extract. **(H)** The standard curve of bilirubin by the HPLC method.

The identification and quantification of chemicals are essential for TCM studies to ensure repeatable and consistent results. According to Chinese Pharmacopoeia and previous reports, liquiritin, baicalin, and paeoniflorin could be representative components for quality control of NHQXW (Zhao et al., 2008; Fan et al., 2019; Wang et al., 2021). *Calculus bovis* (C. bovis) is a key component of NHQXW and bilirubin is the representative compound in C. bovis responsible for its neuropharmacological effects (Yu et al., 2020). In our previous works, baicalin could improve hippocampal neurogenesis in a chronic CORT stress depression mouse model (Gao C. et al., 2018). Hence, we performed bioactive components-based quality control studies of NHQXW using HPLC. As shown in Figures 1A, B, liquiritin, baicalin, and paeoniflorin had good separation and linearity in standard solutions and samples using HPLC analysis. The correlation coefficients indicated a good linear correlation in regression analysis (R^2^ > 0.999) for all constituents in the ranges of 10 μg/mL to 333.3 μg/mL. These results suggest that the method should be reliable with high sensitivity (Figures 1C–E). The content of liquiritin, baicalin, and paeoniflorin was 3.1264 ± 0.0238, 3.0851 ± 0.0255, and 1.0669 ± 0.0058 mg/pill, respectively in NHQXW (3 g per pill). Given that bilirubin has different chemical characteristics from other active compounds, we developed a method to successfully determine the concentration of bilirubin in NHQXW, showing a good linear correlation with R^2^ at 0.9999 using DAD detector (Figures 1F, H), and the content of bilirubin was 4.6326 ± 0.0831 mg/pill in NHQXW.

### 3.2 NHQXW attenuates depressive and anxiety behaviors in mice exposed to chronic restraint and corticosteroid stress

**FIGURE 2:**
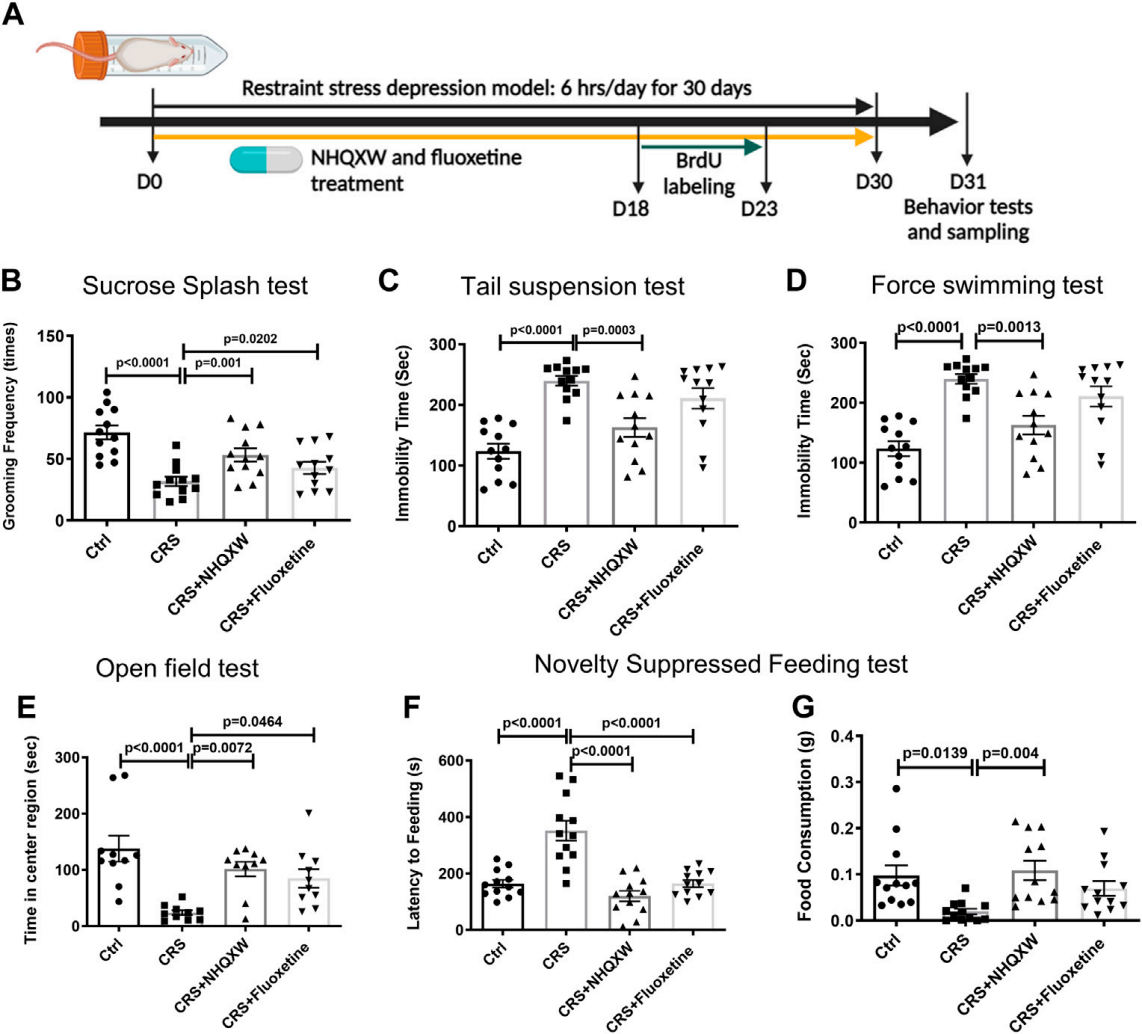
NHQXW treatment attenuated the depressive-like behaviors in chronic restraint stress (CRS) mice. **(A)** The process of restraint stress exposure and drug treatment. The results were presented in 4 groups: vehicle-treated normal control group, restraint stress group, stress plus NQHXW treatment groups at a dosage of 20 mg/kg, and FLX treatment group. The sample size for all groups was 11–12. **(B)** Total grooming time was recorded in 10 min after 10% sucrose splash was sprayed on the back of the mice [F (3, 44) = 11.51, *p* < 0.0001]. **(C)** The immobility time of depressive mice in the tail suspension test [F (3, 44) = 14.24, *p* < 0.0001]. **(D)** The immobility time of depressive mice in the force swimming test [F (3, 40) = 11.33, *p* < 0.0001]. **(E)** Quantification of the time in the center of the open field [F (3, 36) = 9.015, *p* = 0.0001]. **(F, G)** The latency to feeding **(F)** and the food consumption **(G)** were recorded in the novelty-suppressed feeding test of depressive mice [F (3,44) = 21.73, *p* < 0.0001 for latency to feeding; F (3,44) = 5.234, *p* = 0.0035 for food consumption]. Data are presented as mean ± S.E.M.

We first evaluated the anti-depressant activities of NHQXW by using a CRS induced depression mouse model. To induce restraint stress, we placed the mice into a 50 mL polypropylene conical tube for 6 h per day continuously for 30 days. Fluoxetine (FLX) treatment was regarded as the positive control. The first day of the restraint session was recognized as D0. NHQXW or FLX were orally administrated to the mice at D0 and continuously treated for 30 days (Figure 2A). The treatment dosage of NHQXW was 20 mg/g/day, an equivalent dose for human subjects that mimics the clinical use of NHQXW for antidepressant treatment. The TST, FST, OFT, SST, and NSFT were applied to assess the depressive-like behaviors. The restraint stress mice revealed typical anxiety-like behaviors with decreased grooming frequency (Figures 2B–G). In SST, the treatment of NHQXW significantly increased grooming frequency in the restraint stress mice (Figure 2B), the effects of which were similar to the effects of FLX. In the TST and FST, NHQXW treatment significantly attenuated immobility time, the effect of which was also similar to FLX treatment (Figures 2C, D). In the OFT, both the NHQXW and FLX treatment groups spent more time exploring the central field than the vehicle-treated depression mice (Figure 2E). In the NSFT, both the NHQXW treatment group and the FLX group had a shorter latency to food and higher food consumption than the vehicle-treated depressive mice (Figures 2F, G). Taken together, NHQXW attenuated the depressive-like behaviors in the restraint stress mice.

**FIGURE 3:**
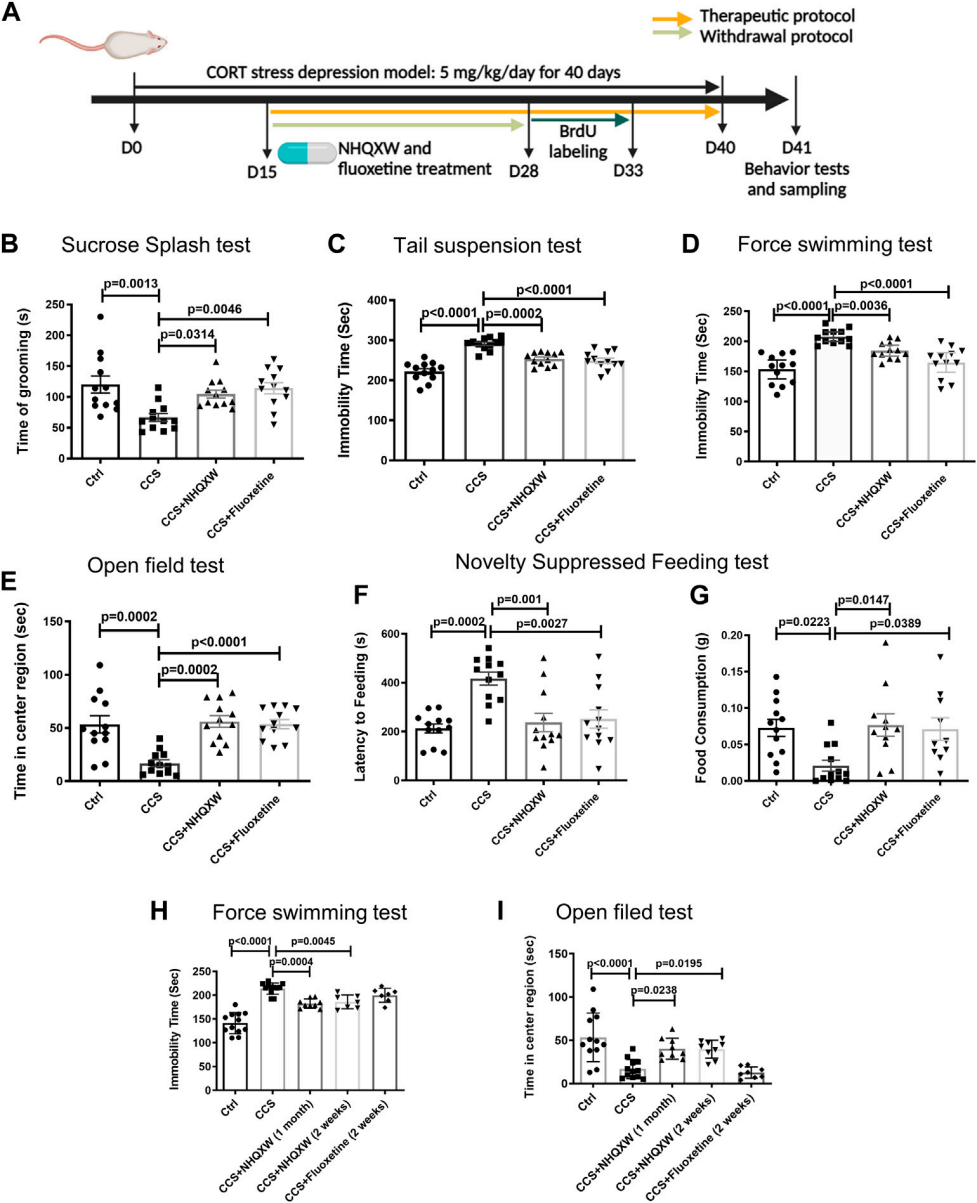
NHQXW treatment attenuated the depressive behaviors in chronic CORT stress (CCS) mice. **(A)** The process of CORT stress exposure and drug treatment with different protocols. The results were presented in 4 groups: vehicle-treated normal control group, CCS group, stress plus NQHXW treatment groups at a dosage of 20 mg/kg, and FLX treatment group. The sample size for all groups was 11–12. **(B)** Total grooming time was recorded in 10 min after 10% sucrose splash was sprayed on the back of the mice [F (3,44) = 6.508, *p* = 0.001]. **(C)** The immobility time of depressive mice in the tail suspension test [F (3,44) = 24.65, *p* < 0.0001]. **(D)** The immobility time of depressive mice in the force swimming test [F (3,44) = 16.73, *p* < 0.0001]. **(E)** Quantification of the time in the center of the open field [F (3,44) = 11.38, *p* < 0.0001]. **(F, G)** The latency to feeding **(F)** and the food consumption **(G)** were recorded in the novelty-suppressed feeding test of depressive mice [F (3,44) = 8.969, *p* < 0.0001 for feeding latency analysis; F (3,41) = 4.608, *p* = 0.0072 for food consumption analysis]. **(H)** The immobility time of depressive mice treated with NHQXW and FLX following withdrawal protocol [F (4,42) = 35.18, *p* < 0.0001]. **(I)** The time in the field center of depressive mice received with withdrawal protocol [F (4,45) = 11.16, *p* < 0.0001]. Data are presented as mean ± S.E.M.

We also assessed the role of NHQXW on CCS mice. As a serotonin reuptake inhibitor, FLX is commonly used for anti-depressive treatment and we also adopted FLX as the positive treatment for chronic stress depressive mice. However, FLX could trigger severe withdrawal reactions in depression treatment (Coupland et al., 1996; Fava et al., 2015). Thus, we further evaluated the anti-depressive effects of NHQXW on the CCS model with both therapeutic and withdrawal protocols. The therapeutic protocol and withdrawal protocol were designed to investigate the anti-depression effects of NHQXW on a short-term and long-term basis (Figure 3A). After 15 days of CORT stress, NHQXW and FLX were administered to the mice for 25 days as the therapeutic strategies. As expected, the CORT-stressed mice showed abnormal depression-like behaviors in different behavior tests including the SST, TST, FST, OFT, and NSFT (Figures 3B–G). The treatment of NHQXW for 30 days significantly attenuated the depressive- and anxiety-like behaviors in the CORT mice in those behavioral tests. Similar to the CRS mice, NHQXW also remarkedly increased the grooming frequency of the CORT mice in the SST (Figure 3B). Furthermore, NHQXW significantly increased the mobility time of the subjected CORT mice in the TST and FST (Figures 3C, D). In the OFT, we recorded the central exploring time in the open field and found that NHQXW and FLX increased the exploring times of the mice in the center of the field (Figure 3E). In the NSFT, we tested the feeling of fear in a novel space. Similar to FLX, the NHQXW treatment significantly ameliorated food consumption and the first latency to the food (Figures 3F, G). We also determined the depressive behaviors with withdrawal protocol. The mice were administrated NHQXW and FLX for 14 days and then these treatments were stopped in the period between day 15 and day 28 until the end of CORT induction. In the FST, NHQXW treatment significantly improved the mobility time in the CORT stress mice even with the withdrawing protocol for 2 weeks (Figure 3H). Similar effects were also found in the OFT. Even after withdrawing NHQXW treatment for 2 weeks, the CORT mice that received NHQXW treatment still revealed an increase in the exploring time in the OFT (Figure 3I). Furthermore, the FLX treatment had no significant effect on the abnormal behaviors in the FST and the OFT (Figures 3H, I). These results indicate that NHQXW had anti-depressive and anti-anxiety effects in the CCS mice. Particularly, NHQXW prevented withdrawal-associated rebound symptoms in the chronic CORT stress mice.

### 3.3 NHQXW promotes hippocampal neurogenesis in both the CRS and CCS depression mouse models

**FIGURE 4:**
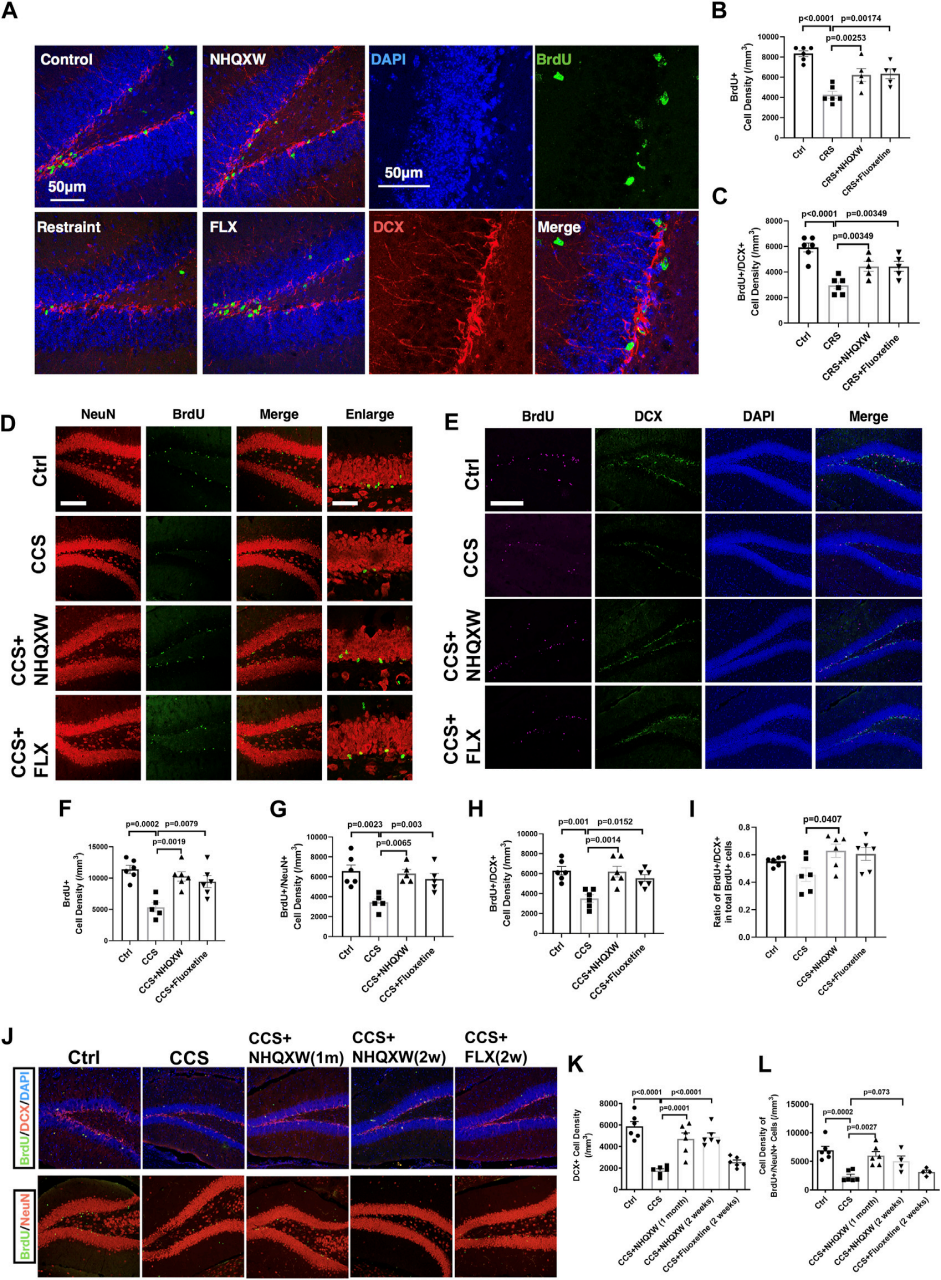
NHQXW promoted neurogenesis in SGZ of two repeat stress depression models. The results were presented in 4 groups: vehicle-treated normal control group, restraint stress group, stress plus NQHXW treatment groups at a dosage of 20 mg/kg, and FLX treatment group. The sample size for all groups was 6. **(A)** Representative confocal image of BrdU and DCX double-positive cells in the hippocampus of the restraint group. **(B, C)** Quantification of BrdU and BrdU/DCX dual positive cell density in SGZ [F (3,18) = 15.79, *p* < 0.0001 for BrdU analysis; F (3,18) = 13.24, *p* < 0.0001 for Brdu/DCX analysis]. **(D)** Representative confocal image of BrdU and NeuN positive cells in the SGZ region. **(E)** Representative immunofluorescence of BrdU and DCX positive cells in the hippocampus. **(F–H)** Quantification of BrdU **(F)**, BrdU/NeuN **(G)** and BrdU/DCX **(H)** dual-positive cells in SGZ [F (3,19) = 10.32, *p* = 0.0003 for BrdU analysis; F (3,17) = 7,474, *p* = 0.0021 for BrdU/NeuN analysis; F (3,20) = 9.087, *p* = 0.0005 for BrdU/DCX analysis]. **(I)** The ratio of BrdU and DCX dual positive cells in total BrdU positive NSCs [F (3,20) = 3.323, *p* = 0.0406]. **(J)** Representative image of BrdU co-staining with DCX and NeuN in SGZ regions. **(K, L)** Quantification of DCX and BrdU/NeuN dual-positive cells in SGZ [F (4,25) = 20.37, *p* < 0.0001 for DCX analysis; F (4,21) = 9.601, *p* = 0.0001 for BrdU/NeuN analysis]. Data are presented as mean ± S.E.M.

We next tracked the effect of NHQXW on modulating the proliferation and differentiation profiles of NSCs in the hippocampus area in both the restraint stress model and the CCS depression mouse model. BrdU was applied to label the proliferation of the NSCs in the hippocampal DG. We performed dual immunofluorescent experiments to co-label the expression of doublecortin (DCX) and neuronal nuclear protein (NeuN) in the region of SGZ to detect the differentiation potentials of endogenous NSCs. The distribution of positive stained NSCs was calculated at the XYZ planes of the confocal image. As such, the double positive staining of BrdU and DCX (BrdU/DCX) was used to identify newborn immature neurons with high differentiation potentials, while BrdU and NeuN dual-positive staining (BrdU/NeuN) was regarded as the newborn mature neurons. The restraint stress model showed significantly decreased BrdU and BrdU/DCX positive populations in the hippocampal area (Figures 4A–C). Treatment with NQHXW markedly elevated the BrdU and BrdU/DCX positive cells, indicating a promising induction of the NSC proliferation and differentiation respectively. The NQHXW treatment had similar effects to FLX in the restraint stress mouse model. Furthermore, the CORT depressive mice also revealed to decrease newborn immature neurons (BrdU/DCX) and newborn mature neurons (BrdU/NeuN) in the hippocampal area, indicating the inhibition of NSCs proliferation and differentiation respectively (Figures 4D, E). Both NHQXW and FLX significantly improved the BrdU/DCX positive cells (Figures 4E, H) and BrdU/NeuN positive cells (Figures 4D, G) in the DG region. Moreover, we also detected the differentiation potential of NSCs in the chronic CORT stress mice (Figures 4J–L). The ratio of BrdU/DCX positive cells in total newborn NSCs was markedly increased compared with the ratio in the CCS mice indicating the differentiation potential of newborn NSCs was improved after NHQXW treatment (Figure 4I). The 2-week NHQXW treatment significantly enhanced the BrdU/DCX and BrdU/NeuN dual-positive cells in the SGZ region of the CORT-treated mice. In contrast, the treatment of FLX for 2 weeks had no remarkable effect on the BrdU/DCX and BrdU/NeuN dual-positive cells in the SGZ region (Figures 4J–L). These results reveal that NHQXW promoted hippocampal neurogenesis in chronic stress mice.

### 3.4 NHQXW upregulates the BDNF/TrkB/ERK/CREB signaling pathway in the hippocampus of the CCS-exposed mice

**FIGURE 5:**
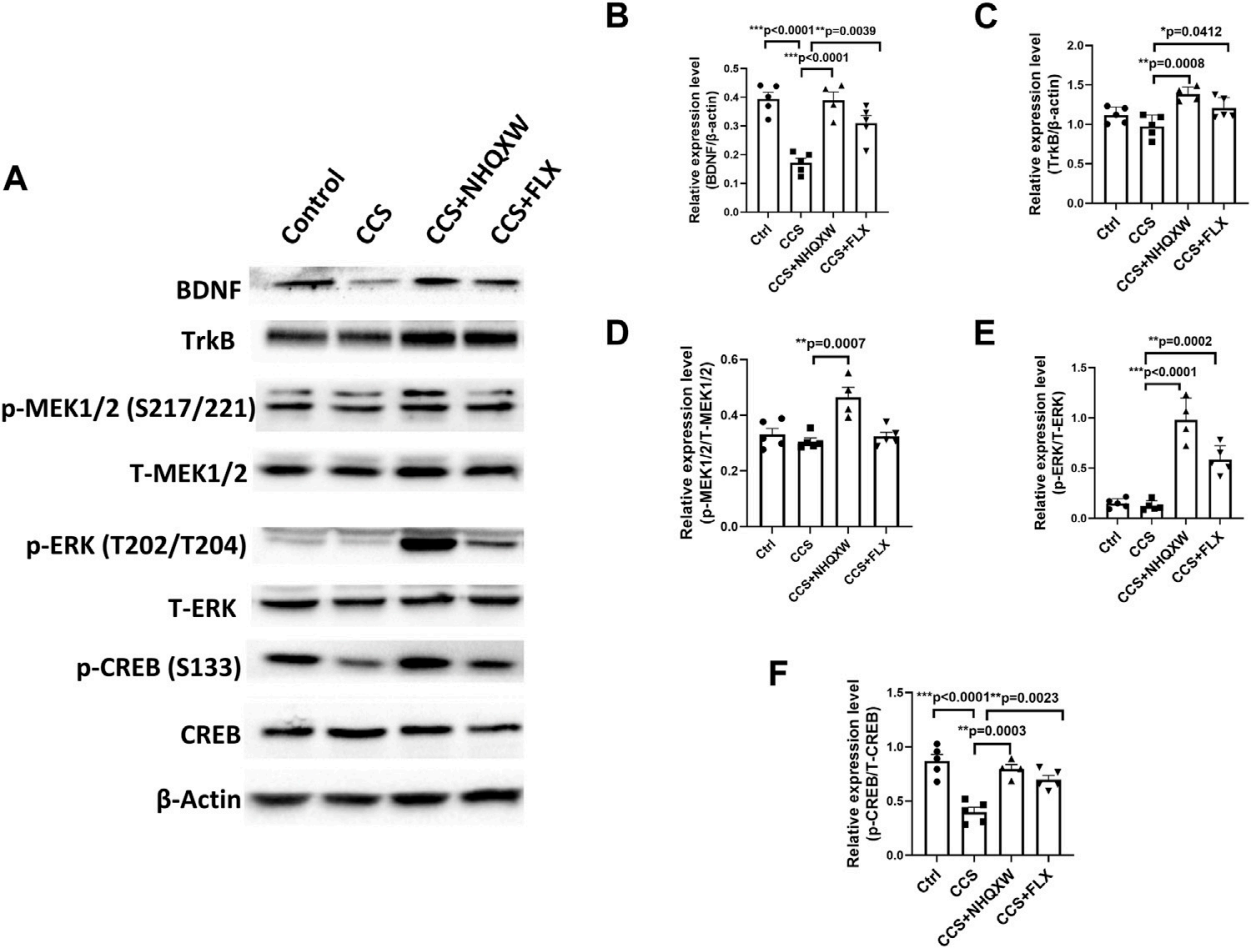
NHQXW regulated the TrkB/ERK/CREB pathway in the hippocampus of CCS mice. The results were presented in 4 groups: vehicle-treated normal control group, CORT stress group, stress plus NQHXW treatment groups at a dosage of 20 mg/kg, and FLX treatment group. The sample size for all groups was 4-5. **(A)** Representative immunoblot results for the expression of BDNF, TrkB, p-ERK (T202/T204), p-MEK1/2 (S217/221), and p-CREB (S133). **(B–F)** Quantification of the relative expression for the ratio of BDNF/β-actin **(B)**, TrkB/β-actin **(C)**, p-MEK1/2 (S217/221)/total MEK1/2 **(D)**, p-ERK (T202/T204)/total ERK **(E)**, and p-CREB (S133)/total CREB **(F)** [F (3,15) = 19.35, *p* < 0.0001 for BDNF analysis; F (3,15) = 8.858, *p* = 0.0013 for TrkB analysis; F (3,15) = 10.38, *p* = 0.0006 for p-MEK (S217/221) analysis; F (3,15) = 45.21, *p* < 0.0001 for p-ERK (T202/T204) analysis; F (3,15) = 18.72, *p* < 0.0001 for p-CREB(S133) analysis]. Data are presented as mean ± S.E.M.

The BDNF-associated signaling pathway plays a crucial role in regulating NSC differentiation for neurogenesis (Martinowich et al., 2007; Brunoni et al., 2008; Bathina and Das, 2015; Miranda et al., 2019). BDNF and its downstream signaling (TrkB, BDNF receptor, and CREB) participate in multiple brain repair aspects including neurogenesis, synaptic plasticity, and synaptic transmission, subsequently affecting depression development (Taliaz et al., 2010; Yu and Chen, 2011). Thus, we explored the effects of NHQXW on the BDNF/TrkB/ERK/CREB signaling pathway in the CCS-exposed mice (Figure 5). After being exposed to chronic CORT stimulation, the vehicle mice revealed the downregulated expression of BDNF, TrkB, p-ERK (T202/T204), p-MEK1/2 (S217/221) and p-CREB (S133) in the hippocampus area (Figures 5A–F). These changes were completely abolished by the treatment with NHQXW (Figures 5A–F). As a positive control, the treatment of FLX also blocked CCS stimulation on the expression of BDNF, p-ERK, and p-CREB but had no effect on p-TrkB in the hippocampus (Figures 5E, F). Taken together, NHQXW could regulate the BDNF/TrkB/ERK/CREB pathway to induce hippocampal neurogenesis in chronic stress-associated depression.

### 3.5 Liquiritin could be a representative compound with neurogenesis-promising effects

**FIGURE 6:**
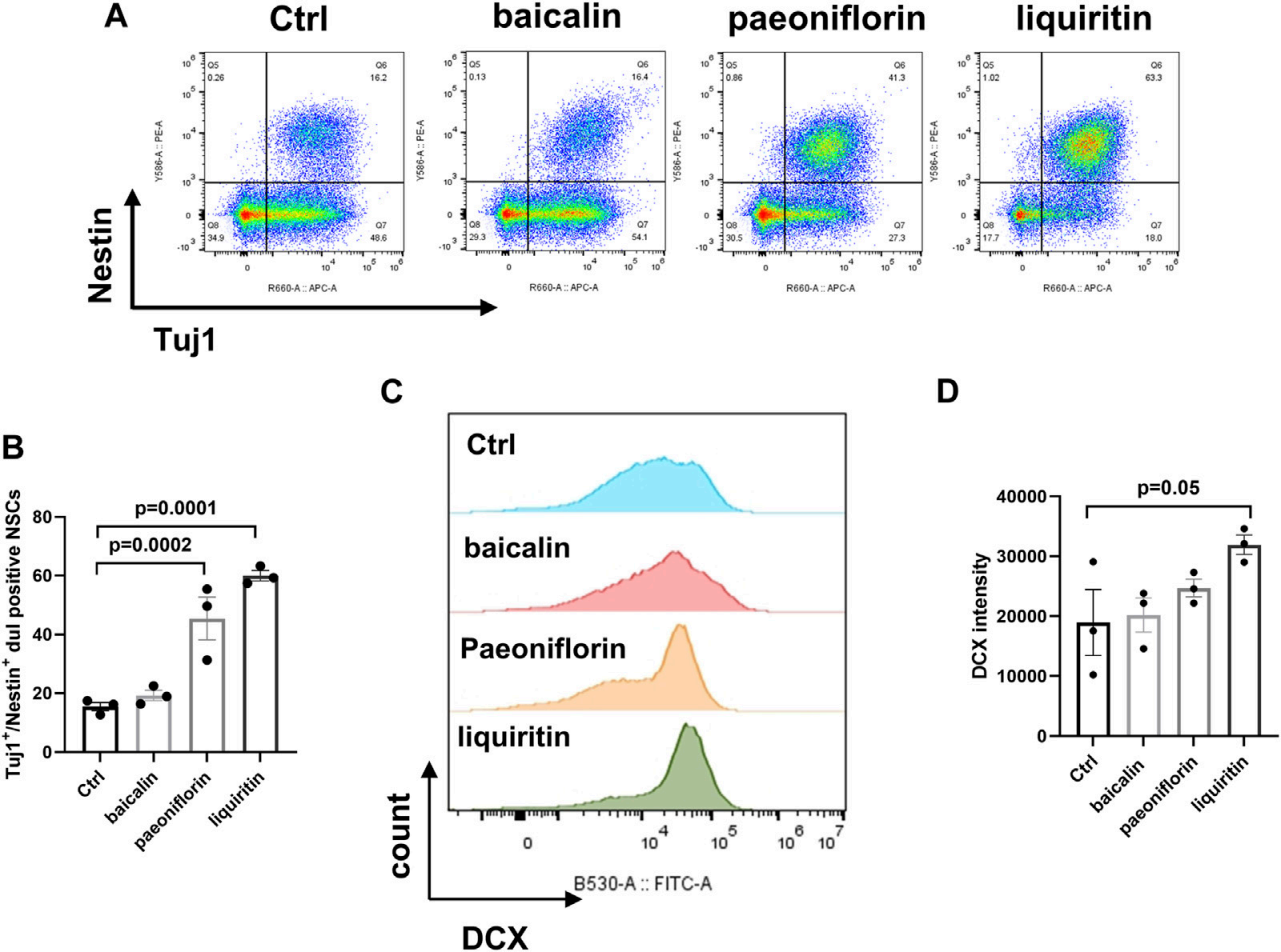
Effects of bilirubin, baicalin, paeoniflorin, and liquiritin as representative active compounds from NHQXW on promoting NSC differentiation. **(A)** Representative flow cytometry results of bilirubin, baicalin, paeoniflorin, and liquiritin on inducing NSC induction. **(B)** Quantification of the Tuj1 and Nestin dual positive population [F (3,8) = 29.88, *p* = 0.0001]. **(C)** Flow cytometry histogram analysis of DCX expression in NSCs. **(D)** Quantification of DCX intensity [F (3,8) = 3.199, *p* = 0.0837]. Data are presented as mean ± S.E.M.

We investigated the bioactivities of paeoniflorin, liquiritin, and baicalin, at a dosage of 10 μg/mL, in promoting neurogenesis by using *in vitro* cultured human NSCs. After 10 days of NSC induction and compound treatment, the expansion and differentiation of NSCs were determined by immunostaining of Nestin/Tuj1 and DCX respectively. The treatments of liquiritin and paeoniflorin significantly increased nestin/Tuj1 positive cells, indicating promising effects on the proliferation (Figures 6A, B). Meanwhile, treatment of liquiritin significantly increased the number of DCX-positive cells, suggesting the promotion of NSC differentiation (Figures 6C, D). Baicalin and paeoniflorin showed no obvious effect on the induction of DCX-positive cells. Furthermore, treatments of baicalin had no effect on the induction of Nestin/Tuj1 and DCX-positive cells. Thus, liquiritin could be a representative ingredient of NHQXW with neurogenesis-promising effects. We then investigated whether the neurogenic effects of liquiritin could be related to the modulation of TrkB, the downstream signaling of BDNF. The CORT stimulation remarkably inhibited the rates of MAP2-positive cells in the cultured NSCs. Furthermore, liquiritin treatment abolished the inhibitory effects of CORT on neurogenesis (Supplementary Figure S1). Furthermore, co-treatment with TrkB inhibitor Cyc-B abolished the effects of liquiritin on the MAP2-positive cells. These results suggest that liquiritin contributes to the effects of NHQXW on the induction of BDNF signaling and the promotion of neurogenesis under CORT stimulation. Thus, liquiritin and paeoniflorin could be used as representative compounds to promote neurogenic efforts, and subsequently they are suitable for use in a bioactivity-guided quantity control study. Of note, baicalin treatment did not show the neurogenesis-promising effects in our *in vitro* results.

## 4 Discussion

**FIGURE 7:**
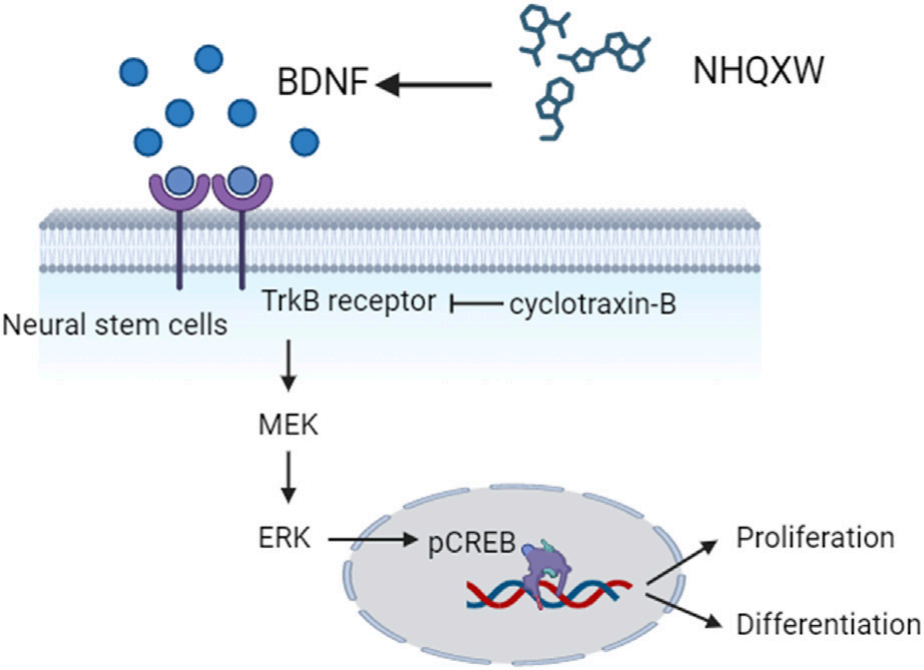
Diagram showing the neurogenic effects of NHQXW on NSCs through the TrkB/CREB pathway.

In the present study, we report that NHQXW ameliorated depressive- and anxiety-like behaviors in both CRS and CCS-induced depressive mouse models. NHQXW revealed anti-depressive effects similar to fluoxetine. The underlying anti-depressive mechanisms could be correlated with the neurogenic bioactivities of NHQXW by activating TrkB/ERK/CREB signaling pathway (Figure 7). With the complex herbal ingredients of NHQXW, we quantitatively analyzed the contents of paeoniflorin, liquiritin, bilirubin, and baicalin as bioactive mark compounds for quality control according to their neurogenesis-promoting activities. The results indicate that NHQXW could be a promising TCM formula to attenuate depressive- and anxiety-like behaviors against chronic stress.

Previous studies by us and others indicate that chronic administration of CORT could continuously exacerbate depression-like behaviors and neurochemical alterations with recurrent episodes in animal models, implying the need to develop therapeutic interventions (Lebedeva et al., 2020). Due to the characterized behaviors and molecular profiles in both restraint stress and CORT stress models (Gregus et al., 2005; Ngoupaye et al., 2018), we selected these animal models to investigate the anti-depressive effects of NHQXW. As expected, the anxiety- and depressive-like behaviors in the restraint stress mice were remarkably attenuated by treatment with NHQXW which had similar effects to FLX. Treatment with NHQXW also inhibited the depressive- and anxiety-like behaviors in the CORT stress mice. With the therapeutic and withdrawal protocols, we further evaluated the anti-depressive effects of NHQXW by using a CCS mouse model. After being treated with NHQXW for 2 weeks, the mice were withdrawn from the NHQXW treatment but were continuously exposed to CORT treatment for 2 weeks. Although the treatment of NHQXW was terminated, the mice still revealed improved mobility time and increased exploring time in the FST and the OFT respectively. However, FLX treatment had no significant effect on the abnormal behaviors in the FST and the OFT. Thus, NHQXW is a promising anti-depressive and anti-anxiety agent that could prevent withdrawal-associated rebound symptoms.

Targeting hippocampal neurogenesis appears to be a promising anti-depression strategy. Previous studies indicate that hippocampal neurogenesis and hippocampal volume sharply decline in depression patients (MacQueen et al., 2003; Videbech and Ravnkilde, 2004). Antidepressants, such as fluoxetine and sertraline, were reported to stimulate neurogenesis in depression therapy (Wang et al., 2008; Anacker et al., 2011). Adult neurogenesis has the potential to integrate into the circuitry of the hippocampus to improve mobility and cognitive behaviors (Aimone et al., 2006). DCX is a commonly used neurogenesis biomarker presented at the stage of neural maturation (Chen et al., 2020). The status of neurogenesis can be identified by detecting the co-immunostaining fluorescence of immature neural biomarker DCX and mature neural biomarker NeuN with exogenous cell tracer BrdU. Thus, we used these biomarkers to investigate the effects of NHQXW on hippocampal neurogenesis in both a restraint stress model and in CORT-induced depressive mice. Both the restraint stress and CORT depressive mice showed significantly decreased hippocampal neurogenesis as detected by BrdU/NeuN and BrdU/DCX staining (Figures 4A–C). Treatment with NQHXW markedly ameliorated the BrdU/NeurN and BrdU/DCX positive cells in the DG region and SGZ region in the animal models. The results reveal that NHQXW could amplify hippocampal neurogenesis in a depression mouse model of chronic stress.

BDNF is a neurotrophic factor that promotes the survival of neural progenitors and improves NSC differentiation into mature functional neurons (Rossi et al., 2006; Wu et al., 2008). BDNF plays a vital role in mental disorders and neurogenesis (Taliaz et al., 2010). TrkB, a receptor of BDNF, triggers cascades of downstream signaling transduction to modulate neurogenesis in the pathogenesis of depression (Li et al., 2008). ERK and CREB are two downstream molecules that participate in the transcription of neurogenic factors (Hao et al., 2004; Gass and Riva, 2007). Given that the profile of NSCs and anxiety-like behaviors were similar in both the restraint stress model and the ORT stress model, we only selected the CORT-treated mice as the representatives for the mechanistic study, saving animal samples for ethical consideration. The results indicate that chronic CORT stimulation downregulated the expression levels of BDNF, TrkB, p-ERK (T202/T204), p-MEK1/2 (S217/221), and p-CREB (S133) in the hippocampus area, and the treatment of NHQXW reserved the changes of those proteins. Therefore, the underlying mechanisms of NHQXW could be related to the regulation of the BDNF/TrkB/ERK/CREB pathway and the improvement of hippocampal neurogenesis as antidepressant bioactivities against chronic stress.

Of note, anxiety-like behaviors have a complicated neuropathological basis with multiple factors and signaling pathways for network regulations. Different from conventional drugs, NHQXW is a TCM formula that contains complex ingredients from the chemistry perspective. Thus, the anti-anxiety and anti-depressive effects of NHQXW should involve complex network regulations with multiple cellular signaling pathways. The BDNF/TrkB/ERK/CREB signaling pathway could be one of the typical cellular signaling pathways affecting hippocampal neurogenesis for the anti-depression effect of NHQXW. Further study is necessary to explore the molecular targets and therapeutic principles of NHQXW for its antidepressant bioactivity. Notably, liquiritin was revealed to promote the induction of NSC proliferation and differentiation whereas paeoniflorin only stimulated the proliferation. However, bilirubin and baicalin had no effect on the induction of proliferation and differentiation. Furthermore, the liquiritin treatment abolished the inhibitory effects of CORT on the development of NSCs into mature neurons. The neurogenesis-promoting effects were associated with the induction of TrkB, the downstream signaling of BDNF. Thus, liquiritin could be one of the representative compounds with neurogenesis-promoting effects. Other active neurogenic and anti-depressant compounds from NHQXW remain to be explored in the future.

Notably, we need to clarify the limitations of this study in several aspects. For mechanistic studies, with the complex chemical and signaling network interactions, the BDNF/TrkB/ERK/CREB signaling pathway is only one of the potential mechanistic pathways affected by NHQXW treatment. We should further investigate other cellular signaling pathways and related active compounds to elucidate the therapeutic principle of NHQXW for antidepressant treatment. For example, neuroinflammation is also a critical process in mental disorders (Kim et al., 2016; Troubat et al., 2021). Proinflammatory cytokines, involving TNFα, IL1β, IL6, and interferon, are important regulators in the pathogenesis of depressive disorder (Simen et al., 2006; Prather et al., 2009; Baune et al., 2010). Besides serotonin reuptake inhibition, anti-inflammatory treatment is one of the other potential therapeutic strategies for depression treatment in clinical settings (Kohler et al., 2016; Miller and Raison, 2016). Currently, there is no relevant study to explore the anti-inflammation bioactivity of the NHQXW formula. Nevertheless, several active compounds contained in NHQXW, such as baicalin, paeoniflorin, and ginsenosides, showed anti-inflammatory effects by inhibiting microglial activation (Lee et al., 2012; Nam et al., 2013; Jin et al., 2019). The anti-inflammation contribution to the anti-depressant effects of NHQXW remains to be further elucidated. With the anti-inflammation bioactivity, NHQXW could also affect glial and microglial functions to affect neurogenesis and depressive and anxiety behaviors, which should be clarified in the future. With the abundant chemicals in the NHQXW formula, our study can only provide very limited insight into understanding the active compounds contributing to its anti-depressant and neurogenesis-promising effects. Even for the selective mark compounds such as paeoniflorin, liquiritin, bilirubin, and baicalin, to confirm their bioactivities in the whole formula, we would need to further investigate their bioavailability, pharmacokinetics, and pharmacodynamics after oral administration of NHQXW. Therefore, we should step forward to further explore the underlying mechanisms and active compounds contributing to the antidepressant and neurogenesis-promising effects of NHQXW, providing solid experimental evidence for well-designed clinical trials in the future.

In conclusion, NHQXW has antidepressant effects against chronic stress-associated depression and its underlying mechanisms could be at least in part correlated with its neurogenesis-promoting effects through activating the BDNF/TrkB/ERK/CREB signaling pathway.

## Data Availability

The raw data supporting the conclusion of this article will be made available by the authors, without undue reservation.
