# Supplementary material for: Niuhuang Qingxin Wan ameliorates depressive-like behaviors and improves hippocampal neurogenesis through modulating TrkB/ERK/CREB signaling pathway in chronic restraint stress or corticosterone challenge mice
Source: Front Pharmacol. 2024 Jan 11;14:1274343. doi: 10.3389/fphar.2023.1274343 (PMC10808638; doi:10.3389/fphar.2023.1274343)
Supplement: Supplementary file 1 [file DataSheet1.docx]

**
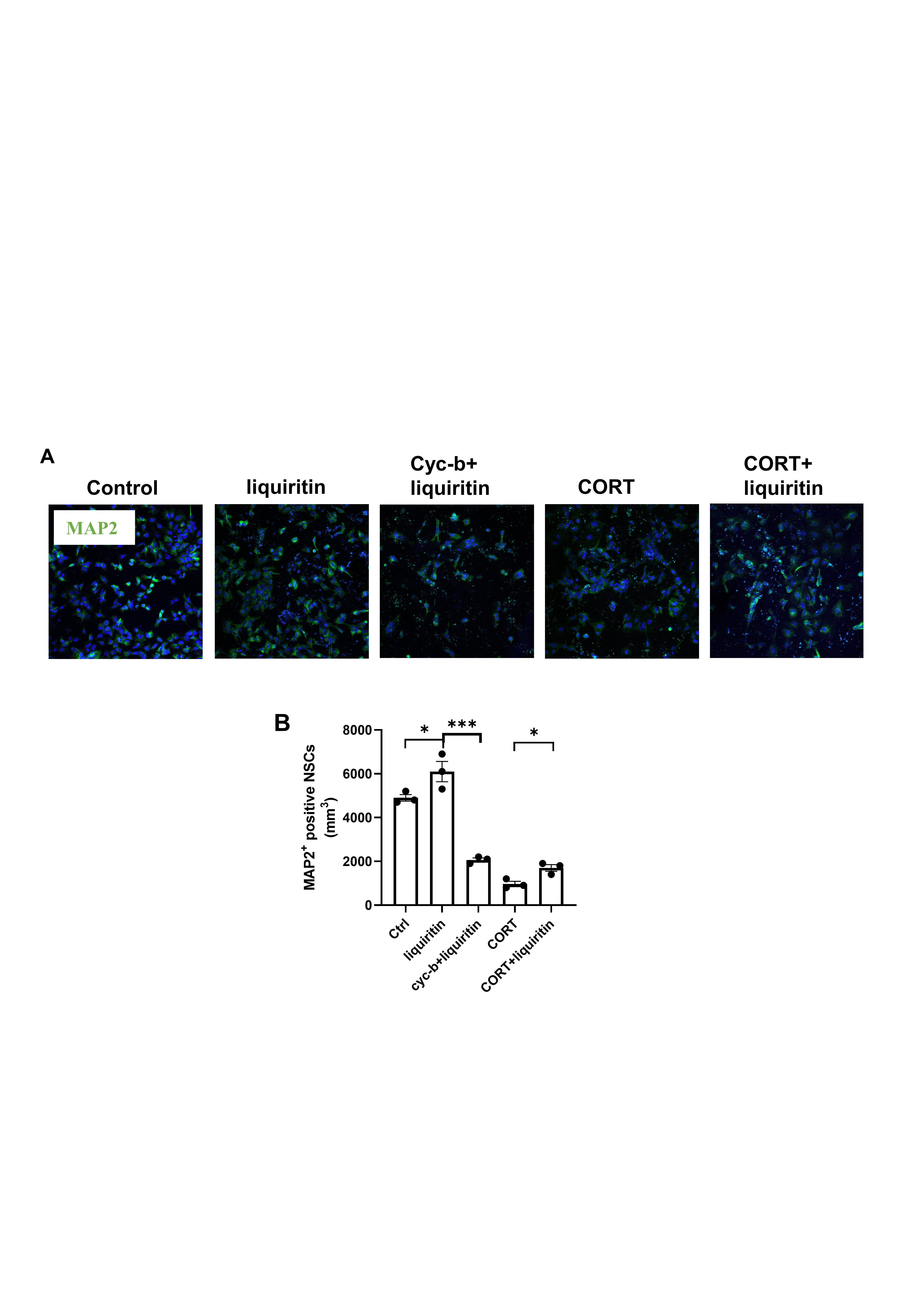
**

**Figure S1. (A)** Representative fluorescence image of MAP2 staining of liquiritin-treated NSC differentiation. **(B)** Quantification of MAP2 positive cells in the image (F(4,10)=87.73). Data are presented as mean ± S.E.M.
